# Antidepressants vs. Placebo for the Treatment of Functional Gastrointestinal Disorders in Adults: A Systematic Review and Meta-Analysis

**DOI:** 10.3389/fpsyt.2018.00659

**Published:** 2018-12-04

**Authors:** Nana Xiong, Yanping Duan, Jing Wei, Ricarda Mewes, Rainer Leonhart

**Affiliations:** ^1^Department of Psychological Medicine, Peking Union Medical College Hospital, Chinese Academy of Medical Science & Peking Union Medical College, Beijing, China; ^2^Department of Psychology, University of Vienna, Vienna, Austria; ^3^Institute of Psychology, University of Freiburg, Freiburg, Germany

**Keywords:** antidepressant, functional gastrointestinal disorder, depression, psychopharmacology, tolerability

## Abstract

**Objective:** To integrate high-quality evidence of the efficacy of antidepressants across different subtypes of functional gastrointestinal disorders (FGIDs).

**Methods:** The Medline, PsycINFO, EMBASE, the Cochrane Library, and Chinese local database were searched up to October 1, 2017. Keywords included all subtypes of FGIDs, antidepressants, and randomized controlled trials (RCTs). We included RCTs with low to moderate risks of bias in comparing antidepressants with placebos as the only intervention in treating adult patients with FGIDs (PROSPERO ID: CRD42015030123). Language was restricted to English or Chinese. Data extraction was independently carried out by two authors, following the Cochrane Handbook for systematic reviews.

**Results:** Of 2,460 records identified, 31 studies reporting on 2,340 participants were included in the meta-analysis. Antidepressants were more effective than placebos in terms of the rate of responder [RR = 1.35 (95% CI 1.12 to 1.63)], and the reduction of target gastrointestinal symptoms [SMD = −0.94 (95% CI −1.33 to −0.54)], and disability severity (moderate effect sizes). Those effects partly remained both at the presence and absence of comorbid depression, and among different subtypes of FGIDs. Subgroup analyses confirmed the benefit of tricyclic and tetracyclic antidepressants, selective serotonin reuptake inhibitors (SSRIs), and trazodone. Efficacy of serotonin and norepinephrine reuptake inhibitors (SNRIs), low doses of antidepressants, and antidepressants in intermediate to long term treatment was inconclusive due to the scarcity of eligible evidence. Compared to placebo, patients on antidepressants reported more adverse events [RR = 1.91 (95% CI 1.23 to 2.96)] and more frequent withdrawal. On average one in 7.4 (95% CI 5.4 to 11.9) patients treated with antidepressants responded, while one in 4.8 (95% CI 3.7 to 6.8) experienced certain adverse effects.

**Conclusions:** Antidepressants were inferior to placebo in terms of tolerability and partly superior regarding efficacy. Before prescribing antidepressants, the benefits and side effects should be carefully evaluated.

## Introduction

Irritable bowel syndrome (IBS), functional dyspepsia (FD), and functional heartburn/functional chest pain (FCP) are the most common subtypes of functional gastrointestinal disorders (FGIDs), now referred as disorders of gut-brain interaction. However, more often than not, different subtypes of FGIDs coexist and overlap (1–3). Patients with FGIDs also shared similar clinical characteristics, such as high levels of comorbid depression and anxiety, impaired social function and health-related quality of life (HRQoL), abuse history, and increased outpatient services, surgeries, physician visits and healthcare costs (4). For example, the rates of comorbidity with anxiety and depression in patients with FGIDs were estimated as 34.3–54.2% (3, 5–7). A study based on a large community sample in Norway also supported a linear relationship between functional somatic symptoms and depression or anxiety (8). Furthermore, common pathophysiological mechanisms were shared by most FGIDs, including motor dysfunction, visceral hypersensitivity, central nervous system dysregulation, altered mucosal immune function, and imbalanced intestinal microbiota (9, 10).

Given consideration to the variations in severity of FGIDs, stepped care approaches are recommended, with interventions ranging from watchful waiting to symptomatic relief to multimodal treatment (11). Among them, antidepressants were used especially for those with poor response to conventional medical therapies to ease pain, constipation, or dyspeptic symptoms (including acid-suppressive drugs such as proton-pump inhibitors (PPIs) or histamine H2-receptor antagonists, prokinetic agents, bulking agents, and antispasmodics medications), and with coexisting impairment of mood (12, 13). Nevertheless, evidence yielded from those studies was contradictory. Systematic reviews and clinical guidelines before 2008 basically found no clear evidence of benefit for antidepressants (14–16), while updated reviews generally indicated that they were effective for the treatment of IBS (17, 18), FD (10), FCP (19), and functional abdominal pain (20). However, unlike the real world, most researches only focused on one specific subtype of FGIDs, while the comorbidity with other subtypes of FGIDs and mental disorders often was not accounted for. The only meta-analysis investigating the effectiveness of antidepressants across different subtypes of FGIDs included patients with FD, IBS or both (21). Even though results showed a favorable outcome, the author pointed out that they were markedly influenced by one study that did not actually fulfill the criteria necessary to be included, and that the overall quality of included studies was low-to-moderate. In addition, answers to a series of important clinical questions are still lacking. For example, will FGIDs patients without mental disorders (especially depression and anxiety spectrum disorders) benefit from antidepressants treatment, is any kind of antidepressants superior to another in treating FGIDs, and whether antidepressants treatment at low dosages and short durations enough for patients with FGIDs or not?

Therefore, a systematic review and meta-analysis encompassing the most recent high-quality studies is highly needed to guide the administration of antidepressant medication across different subtypes of FGIDs. Our objectives were to assess the efficacy and tolerability of antidepressant medication compared with placebo in randomized controlled trials (RCTs) for FGIDs in adults. In a second step, subgroup analyses were performed to explore whether the specific syndrome, types and dosages of antidepressants, the comorbidity of depression, and the duration of treatment affect efficacy.

## Methods

The review was performed according to the Preferred Reporting Items for Systematic Reviews and Meta-Analyses (PRISMA) statement (22) and the recommendations of the Cochrane Collaboration (23). The PROSPERO ID of this systematic review's protocol is CRD42015030123.

### Study Design, Participants, Interventions, and Comparators

We included published RCTs evaluating the efficacy of antidepressants in treating adult patients 18 years of age or older with FGIDs. Because of the changes in diagnostic classifications over time, there was no special requirement for the diagnostic criteria of FGIDs as long as the definitions used were clearly described. Still, the diagnostic criteria adopted in these studies were noted for post analysis and comparison. Treatment groups had to have received antidepressants regularly at any dose as the only intervention. All types of antidepressants were eligible for this review, including tricyclic antidepressants (TCAs), tetracyclic antidepressants (TeCAs), monoamine oxidase inhibitors (MAOIs), selective serotonin reuptake inhibitors (SSRIs), serotonin and norepinephrine reuptake inhibitors (SNRIs), and serotonin antagonist and reuptake inhibitor (SARI). Mirtazapine was included in the TeCA group according to the similar chemical structure, even though it is sometimes described as an atypical antidepressant and a noradrenergic and specific serotonergic antidepressant (NaSSA). Herbal medications such as St John's wort were excluded since they were non-standard antidepressants. Studies were also classified into two subgroups according to the dosages of antidepressants: adequate vs. smaller dosages (no ultra-dosage was identified), based on the target range defined by the U.S. Food and Drug Administration (FDA)-approved labeling. We excluded studies that combined antidepressants with other drugs with possible effects, or with any type of psychotherapy or exercise program. Comparison groups had to have received a placebo having an appearance identical to the antidepressants. Studies with treatment as usual or with other possible active drugs or non-drug treatments as control conditions were also excluded.

Besides, the meta-analysis excluded trials with an observation period <4 weeks since the effect of antidepressants could not be established. Articles with high risks of bias were also excluded from the meta-analysis (see study quality evaluation below).

### Data Sources and Search Strategy

The Medline, PsycINFO, EMBASE, the Cochrane Library, and Chinese database (including Sinomed, China National Knowledge Infrastructure (CNKI), and WanFang data) were systematically screened up to October 1, 2017.

We combined the extended search term of all subtypes of FGIDs, antidepressive agents, and RCTs (see the supplementary search strategies). Language was restricted to English or Chinese. Reference lists from reviewed articles were manually searched to identify relevant articles. In addition, authors of eligible RCTs were invited to provide further data if there's no extractable data reported.

### Study Selection

Two authors (NX and YD) independently screened each title and abstract to evaluate papers in terms of their relevance to the review, and assessed the methodological quality and potential bias of relevant articles by the tools developed by the Cochrane collaboration (23). The reviewers met every week to reach an agreement about the inclusion or exclusion of articles. If necessary, a senior investigator (JW) was consulted.

### Study Quality Evaluation

According to the recommendations of the Cochrane Collaboration (20), the risk of bias was assessed from eight aspects: random sequence generation (selection bias), allocation concealment (selection bias), blinding of participants and of personnel/care providers (performance bias), blinding of outcome assessor (detection bias), incomplete outcome data (attrition bias), selective reporting (reporting bias), group similarity at baseline (selection bias), and other bias (funding bias). We explicitly judged each of these eight criteria as exemplifying a low, high, or unclear risk of bias. We labeled a study as high quality (low risk of bias) if it met at least six of the eight validity criteria, moderate quality (moderate risk of bias) if it met three to five criteria, and low quality (high risk of bias) if it met less than three of the eight validity criteria. Articles of low quality were excluded from the meta-analysis.

### Data Extraction

Data extraction was then independently carried out by the two reviewers (NX and YD), using standardized forms. Data from intention-to-treat (ITT) analysis was preferred when it was reported together with per-protocol analysis. For studies with one control group and two antidepressants, the control group was split into two equal groups for comparison. For studies with a cross-over design, only data before the wash period and the cross-over was synthesized into the meta-analysis to avoid the potential carry-over effect. For articles containing *post-hoc* analyses of the same study, data were combined into one data set. Continuous outcomes were analyzed using the number of participants, the mean, and the standard deviation (STD). Missing STD values were calculated from reported confidence intervals (CI), standardized errors (SE), or *p*-values. Data was estimated from figures if unavailable from tables or the text. When none of the above data were reported, baseline STD or the highest STD from all studies reviewed for the same variable and group were used.

### Measures of Treatment Effects

Primary outcomes included the rate of responder, the reduction of target gastrointestinal symptoms, and disability reduction, which were measured in the following ways:

1. The *responder* was a dichotomous outcome variable measuring overall clinical efficacy. Depending on the trials reviewed, the definitions of a responder were close with that of either the response rate or the remission rate. Reports based on unclear definition or definition less restrict than the common response rate (at least 50% reduction of symptoms) were ruled out. As a result, outcomes from 16 studies (18 comparisons) were synthesized, which defined the responders as patients with (1) more than a 50% reduction from initial symptom score or number of days with symptoms (*n* = 5); (2) a loss of all symptoms or scores below case level (*n* = 5); (3) a self-reported adequate relief of symptoms (*n* = 5); or (4) high satisfaction with the treatment (*n* = 1), respectively.
2. The *reduction of target gastrointestinal symptoms* was a continuous outcome variable, reported as the symptom score or its reduction at the end-point. In summary, tools used by studies reviewed were: the visual analog score (VAS), the self-rated severity of a target symptom on a five-point scale, the composite symptom index of intensity multiplied by frequency, the number of symptoms among major symptoms addressed by the Rome criteria, the Hopkins Symptom Check List (SCL-90-R), the physician-rated clinical global impression of illness severity (CGI-S), the McGill Pain Questionnaire, Patient Assessment of Upper Gastrointestinal Symptom Severity Index (PAGI-SYM), dyspepsia symptom severity (DSS) questionnaire, Nepean Dyspepsia Index (NDI), Hong Kong dyspepsia index (HKDI), and Bowel Symptoms Severity Rating Scale (BSSRS).
3. The *disability* reduction was also a continuous outcome, represented by either the severity of disability or by the impairment to HRQoL. Measures employed by studies reviewed were the sickness impact profile (SIP), the VAS, Sheehan's disability scale (SDS), the European quality of life−5D scale (EQ-5D), the 36-item short form health survey (SF-36), and the IBS-QOL.

For the purposes of this meta-analysis, at least one of these three outcomes had to be reported by all studies included. Secondary outcomes included withdrawal from trials due to adverse events, lack of improvement, non-adherence to treatment, being lost to follow-up, or any other reason, and the occurrence of patients with adverse events (AEs). Tolerability was assessed and represented by the proportion of patients who withdrew because of adverse events.

### Statistical Procedure

#### Data Analysis

Statistical heterogeneity was tested using the I^2^ statistic and chi-square test (24) (significant level: I^2^ > 50% and *p* < 0.1). The random effects model was used for heterogeneous data while the fixed-effects model was used for data without significant heterogeneity. For primary outcomes with significant heterogeneity, meta-regression analyses were conducted to explore potential moderators, among subtypes of FGIDs, classes of antidepressants, comorbidity with depression, treatment duration and funding bias, to account for it.

Because of the variability in measurements across studies, effect size (ES) was reflected by standardized mean difference (SMD) for continuous outcomes, using the Hedges' g method. An ES >0.8 was considered as large, between 0.6 and 0.8 moderate, and between 0.2 and 0.6 small (25). For dichotomous outcomes, risk ratios (RR) were pooled with 95% confidence intervals (CIs). The number need to treat (NNT) and number need to harm (NNH) were calculated accordingly. The likelihood of significant publication bias was assessed through Begg's funnel plot (26) and by testing for asymmetry using Egger's test statistic (27).

The RevMan 5.2 (28) and the software package Stata (15.0) were used for statistical analyses.

#### Subgroup Analyses

Subgroup analyses were planned as a priori to investigate potential moderators influencing our primary outcomes, including the subtypes of FGIDs, types and dosages of antidepressants, comorbidity with depression, and treatment duration.

#### Sensitivity Analyses

Sensitivity analyses were conducted to test the reliability of results, which were performed by excluding studies with approximated data and studies from China.

## Results

### Characteristics of Included Studies

**Figure 1:**
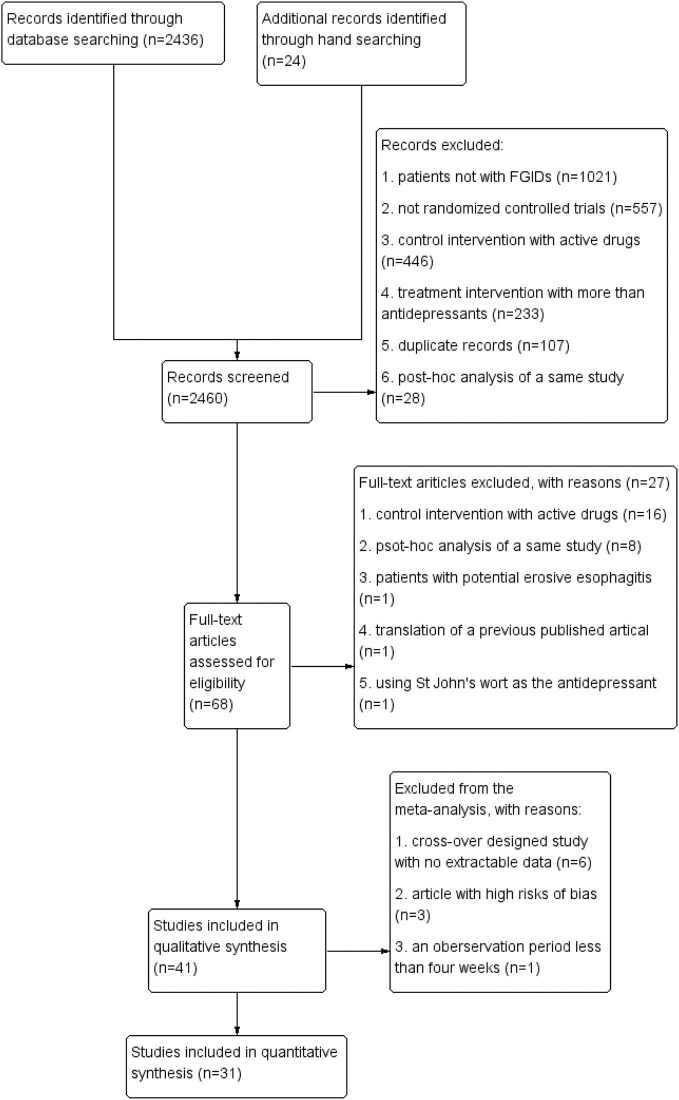
Flow chart.

Overall, 2,460 records were identified. Among them, 41 studies met the inclusion criteria for the review, and 31 of them were included in the meta-analysis (flow chart, see Figure 1). Data from six articles were not synthesized into the meta-analysis due to the lack of extractable data before the wash period and the cross-over (29–34), three articles due to their high risks of bias (35–37), and one article due to the observation period of 3 weeks only (38).

Thirty-one RCTs with a total of 2,340 participants were included in the meta-analysis, with 69.8% being women (range 12.9–100%). Treatment duration lasted 9.6 weeks on average (range 4–34). Only two study published in Chinese was included (39), and the remaining studies were published in English.

#### Study Quality

**Figure 2:**
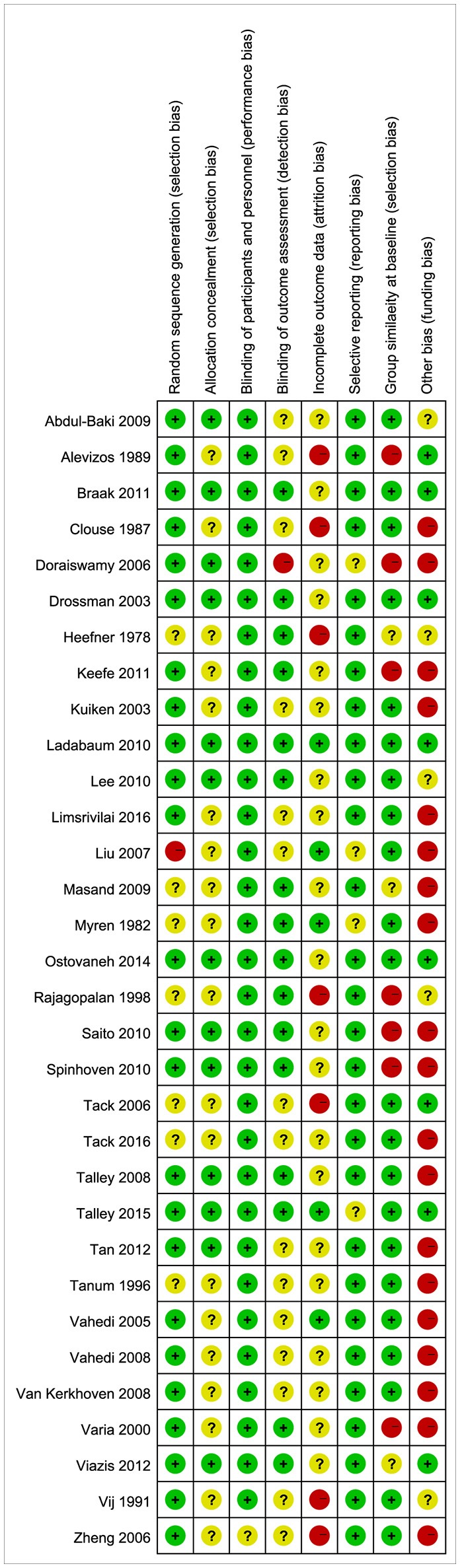
Risk of bias summary: risk of bias item for each included study.

Based on the Cochrane criteria, eight studies (25.8%) had a high quality while 23 studies (74.2%) had a moderate quality. Funding bias (54.8%), selection bias (25.8%), and attrition bias (25.8%) were the most common risks of bias (see Figure 2).

#### Subgroups of FGIDs

Among 31 studies included, ten focused on functional esophageal disorder (FED) [defined as non-cardiac chest pain (*n* = 5), functional chest pain (*n* = 1), and functional heartburn or hypersensitive esophagus (*n* = 4)] (39–48), six on FD (49–54), 13 on IBS (55–67), and two on mixed types (functional bowel disorder and FGID, respectively) (68, 69).

The Rome criteria were used in 16 out of the 31 studies, mostly Rome II criteria (*n* = 10). Seven other studies defined their subjects as suffering from persisting dyspeptic symptoms or bowel disturbance with no structural esophageal abnormality. Five studies of non-cardiac chest pain requested chest pain and a normal coronary angiogram or stress test. One each adopted the Thompson and Heaton's criteria, the definition of “irritable colon syndrome,” and diagnosis confirmed by a gastroenterologist, respectively.

#### Types and Dosages of Antidepressants

Thirty-three comparisons of antidepressants vs. placebo were carried out (see Supplementary Table 1). Among them, 12/33 (36.4%) used TCAs; 2/33 (6.1%) used TeCAs; 15/33 (45.5%) used SSRIs; two SNRIs (venlafaxine); and two SARI (trazodone). Generally, the doses of antidepressants were considered adequate to treat depression except for four studies with lower doses [amitriptyline 10 mg (65), imipramine 25 mg (44), trimipramine 25 mg (60), and trazodone 50–100 mg (50) daily, respectively].

#### Comorbid Depression

Eleven studies excluded patients with depression based on different criteria. Among them, four studies excluded participants based on diagnostic interviews (63, 70) or the evaluation of a study psychiatrist (41, 47). Seven studies excluded participants based on the results of depression screeners, including the Hamilton Depression Scale, the Zung Self-rating Depression Scale, the Beck Depression Inventory, and others. The other 20 studies did not specifically exclude persons with depression and probably include both patients with and without depression. Thus, the subgroup analyses could at best be classified as with depressive patients excluded and included. No subgroup of patients all with depression could be established.

### Effects of Antidepressant Medication

#### Primary Outcomes

##### Rate of responder at post-treatment

As summarized in Table 1, in general, antidepressants were superior to placebo [RR = 1.35 (95% CI 1.12 to 1.63), I^2^ = 50%], resulting in a NNT of 7.4 (95% CI 5.4 to 11.9).

**Table 1:** Efficacy of antidepressants in subgroups of patients with functional gastrointestinal disorders.

**Outcomes**	**Subgroups**	**Number of arms**	**Number of participants**	**RR/SMD (95% CI)**
Rate of responder	Total	17	1,455	1.35 (1.12, 1.63)
	**SUBTYPES OF FGID**			
	FED	5	329	1.72 (1.18, 2.53)
	FD	4	603	1.06 (0.86, 1.31)
	IBS	6	312	1.28 (0.90, 1.82)
	Mixed	2	211	2.13 (0.64, 7.09)
	**TYPES OF ANTIDEPRESSANTS**			
	TCAs	7	625	1.34 (1.12, 1.61)
	TeCAs	1	47	4.18 (1.68, 10.4)
	SSRIs	7	594	1.26 (0.87, 1.84)
	SNRIs	1	160	0.97 (0.65, 1.44)
	SARI	1	29	1.68 (0.74, 3.80)
	**DOSAGES OF ANTIDEPRESSANTS**			
	Adequate	15	1,318	1.36(1.10, 1.68)
	Smaller	2	137	1.32(0.77, 2.28)
	**DEPRESSION**			
	Excluded	4	154	1.23 (0.62, 2.45)
	Included	13	1,525	1.39 (1.15, 1.67)
	TREATMENT DURATION^*^			
	5–8 weeks	10	824	1.45 (1.08, 1.96)
	9–12 weeks	7	663	1.20 (1.00, 1.44)
	More than 12 weeks	1	75	1.85 (1.09, 3.12)
Reduction of GI symptoms	Total	28	1,961	−0.94 (−1.33, −0.54)
	**SUBTYPES OF FGID**		
	FED	9	523	−1.08 (−2.17, 0.02)
	FD	7	798	−0.65 (−1.20, −0.10)
	IBS	10	392	−1.13 (−1.76, −0.49)
	Mixed	2	248	−0.52 (−1.99, 0.95)
	**TYPES OF ANTIDEPRESSANTS**			
	TCAs	9	662	−0.36 (−0.94, 0.23)
	TeCAs	2	81	−1.36 (−1.85, −0.87)
	SSRIs	13	899	−1.18 (−1.82, −0.53)
	SNRIs	2	210	−0.97 (−3.35, 1.41)
	SARI	2	109	−1.75 (−2.76, −0.74)
	**DOSAGES OF ANTIDEPRESSANTS**			
	Adequate	24	1,683	−0.99 (−1.39, −0.60)
	Smaller	4	278	−0.53 (−2.18, 1.13)
	**DEPRESSION**			
	Excluded	11	671	−0.99 (−1.55, −0.42)
	Included	17	1,290	−0.90 (−1.45, −0.35)
	TREATMENT DURATION^*^			
	1–4 weeks	13	712	−1.57 (−2.33, −0.80)
	5–8 weeks	18	1,155	−0.84 (−1.34, −0.35)
	9–12 weeks	9	715	−0.61 (−1.12, −0.90)
	More than 12 weeks	1	58	−0.47 (−1.00, 0.05)
Reduction of disability severity	Total	8	274	−0.78 (−1.28, −0.28)
	**SUBTYPES OF FGID**			
	FED	1	58	−0.16 (−0.68, 0.35)
	FD	1	34	−2.24 (−3.12, −1.36)
	IBS	5	143	−0.72 (−1.25, −0.19)
	Mixed	1	39	−0.37 (−1.01, 0.26)
	**TYPES OF ANTIDEPRESSANTS**			
	TCAs	2	57	−0.59 (−1.29, −0.11)
	TeCAs	2	73	−1.28 (−3.11, 0.55)
	SSRIs	4	144	−0.62 (−1.26, 0.02)
	**DOSAGES OF ANTIDEPRESSANTS**			
	Adequate	8	274	−0.78 (−1.28, −0.28)
	Smaller	0	0
	**DEPRESSION**			
	Excluded	4	134	−1.18 (−2.14, −0.23)
	Included	4	140	−0.36 (−0.71, −0.02)
	TREATMENT DURATION^*^			
	1–8 weeks	6	223	−0.74 (−1.50, 0.02)
	More than 8 weeks	3	109	−0.43 (−0.90, 0.04)
Improvement of HRQoL	Total	9	1,019	0.36 (0.08, 0.64)
	**SUBTYPES OF FGID**			
	FED	2	113	1.15 (0.66, 1.63)
	FD	4	603	0.19 (−0.05, 0.44)
	IBS	2	102	0.12 (−0.92, 1.16)
	Mixed	1	201	0.25 (−0.04, 0.55)
	**TYPES OF ANTIDEPRESSANTS**			
	TCAs	4	487	0.62 (0.17, 1.07)
	SSRIs	4	372	0.21 (−0.21, 0.62)
	SNRIs	1	160	−0.06 (−0.37, 0.25)
	**DOSAGES OF ANTIDEPRESSANTS**			
	Adequate	8	936	0.23 (0.02, 0.44)
	Smaller	1	83	1.33 (0.85, 1.81)
	**DEPRESSION**			
	Excluded	4	368	0.35 (−0.09, 0.69)
	Included	5	651	0.41 (−0.01, 0.84)
	**TREATMENT DURATION**			
	1–8 weeks	5	468	0.32 (−0.23, 0.88)
	More than 8 weeks	4	551	0.38 (0.20, 0.56)

* *Since outcomes at different endpoints could be reported more than once from one study, the total number of comparisons was larger than that of studies. FD, functional dyspepsia; FED, functional esophageal disorders; FGID, functional gastrointestinal disorders; IBS, irritable bowel syndrome; SARI, serotonin antagonist and reuptake inhibitor; SNRIs, serotonin and norepinephrine reuptake inhibitors; SSRIs, selective serotonin reuptake inhibitors; TCAs, tricyclic antidepressants; TeCAs, tetracyclic antidepressants*.

##### Reduction of target symptoms at post-treatment

Antidepressants were more effective in reducing the target gastrointestinal symptoms in the overall population [SMD = −0.94 (95% CI −1.33 to −0.54), I^2^ = 94%].

##### Reduction of disability at post-treatment

The combined data indicated that antidepressants were superior to placebo in reducing disability severity [SMD = −0.78 (95% CI −1.28 to −0.28), I^2^ = 72%], as well as improving HRQoL [SMD = 0.36 (95% CI 0.08 to 0.64), I^2^ = 77%].

For all primary outcomes reported above, the meta-regressions were carried out to explore the potential moderating impact of each potential variable. However, results indicated that no significant moderating impact was found among potential variables of types of FGIDs, classes and dosages of antidepressants, comorbidity with depression, and treatment duration (all *p* > 0.05), which meant none of them had significant influence on the primary outcomes.

#### Secondary Outcomes

##### Withdrawal from trials

Twenty-eight comparisons with 2,011 participants were entered into the analysis of withdrawal due to any reason. Altogether, the risk of withdrawal from the trial was higher in patients taking antidepressants [RR = 1.33 (95% CI 1.12 to 1.57), I^2^ = 0%]. The risks of withdrawing due to adverse effects [RR = 2.40 (95% CI 1.73 to 3.32), 22 comparisons, 1,553 participants], and other reasons including study termination on subject's request [RR = 2.42 (95% CI 1.04 to 5.65), 7 comparisons, 725 participants] were also higher in patients taking antidepressants. There was no significant difference between patients taking antidepressants and those taking placebo regarding withdrawal due to lack of efficacy [RR = 0.73 (95% CI 0.46 to 1.14), 7 comparisons, 633 participants], non-adherence to the treatment protocol [RR = 0.68 (95% CI 0.39 to 1.19), 3 comparisons, 352 participants], and being lost to follow up [RR = 0.68 (95% CI 0.38 to 1.19), 9 comparisons, 993 participants].

##### Occurrence of patients with adverse events

Two ways were adopted by studies to report the occurrence of AEs: the frequency of AEs, and the number of patients with AEs. For those reported the frequency, the total number often outweighed the number of patients included, which made the data synthesis and comparison impossible. Therefore, only studies that reported the number of patients with AEs were included in the analysis. As a result, eleven studies with 896 participants were included. Compared to patients on placebo, patients on antidepressants had a higher relative risk of experiencing side effects [RR = 1.91 (95% CI 1.23 to 2.96)]. The number needed to be harmed was 4.8 (95% CI 3.7 to 6.8). Classified by the types of antidepressants, no difference was detected between TCAs [RR = 1.44 (95% CI 0.98 to 2.11)] and SSRIs [RR = 1.33 (95% CI 0.85 to 2.09)] in terms of the occurrence of patients with AEs (*p* = 0.80, I^2^ = 0%).

According to all 31 studies that reported the AEs and the frequency, nausea, sedation, dizziness, dry mouth, nervousness, diarrhea or constipation, headache, somnolence or insomnia, and abnormal ejaculation were the most frequently reported by patients taking antidepressants. Serious adverse events were only reported by patients with IBS treated by amineptine: one developed jaundice and one had urine retention and aggravation of pre-existing glaucoma. No other serious adverse events or patients who died in the included studies were reported.

### Subgroup Analyses

#### Subtypes of FGIDs

No significant difference was detected among different subtypes of FGIDs regarding both the rate of responder (*p* = 0.12, I^2^ = 48.0%) and the reduction of target gastrointestinal symptoms (*p* = 0.66, I^2^ = 0%). The HRQoL of patients with functional esophageal disorders was better improved (*p* < 0.01, I^2^ = 76.1%), and the disability severity was more reduced in FD and IBS patients (*p* < 0.01, I^2^ = 82.2%; See detailed effect size in Table 1).

#### Types of Antidepressants

The difference among the rate of responder of five types of antidepressants was marginally significant (*p* = 0.05, I^2^ = 55.1%). According to the effect size, as summarized in Table 1, TCAs [1.34 (95% CI 1.12 to 1.61)] and TeCAs [4.18 (95%CI 1.68 to 10.42)] showed a significantly higher rate of responder than placebo, while the results of other types of antidepressants were inconclusive.

Marginal significant difference was also detected in reducing the target gastrointestinal symptoms among five types of antidepressants (*p* = 0.05, I^2^ = 54.9%). Among them, TeCAs, SSRIs, and SARI were superior to placebo.

No significant difference was found among different types of antidepressants in terms of decreasing disability severity (*p* = 0.78, I^2^ = 0%) or improving HRQoL (*p* = 0.06, I^2^ = 66.6%).

#### Dosages of Antidepressants

With limited data, no significant difference was detected between antidepressants with adequate and smaller dosages to treat depression in terms of rate of responder (*p* = 0.93, I^2^ = 0%) and symptom reduction (*p* = 0.59, I^2^ = 0%). However, the effect size indicated that the efficacy of antidepressants with smaller dosages was inconclusive (see Table 1). Data from one study showed that the HRQoL was better improved by a lower dosage of antidepressant (*p* < 0.01, I^2^ = 94.1%).

#### Comorbidity With Depression

The rate of responder (*p* = 0.74, I^2^ = 0%), the reduction of main gastrointestinal symptoms (*p* = 0.83, I^2^ = 0%), and the improvement of disability (*p* = 0.11, I^2^ = 60.0%) and HRQoL (*p* = 0.70, I^2^ = 0%) did not differ significantly no matter when FGID patients with depression were excluded or not, even though the effect size of the rate of responder for FGID patients without depression was inconclusive [RR = 1.23 (0.62, 2.45), see Table 1].

#### Treatment Duration

At different endpoints, no significant difference was detected between antidepressants and placebo in terms of the rate of responder (*p* = 0.22, I^2^ = 33.7%), reduction of the target symptoms (*p* = 0.12, I^2^ = 49.3%), reduction of disability severity (*p* = 0.49, I^2^ = 0%), and improvement of HRQoL (*p* = 0.85, I^2^ = 0%). However, only two RCTs included have followed up participants for more than 12 weeks.

### Sensitivity Analyses

Among 29 comparisons concerning the reduction of target gastrointestinal symptoms, seven comparisons were estimated from graphs or studies within the subgroup. The results for the overall efficacy still favored antidepressants (SMD = −0.86 [95% CI −1.30 to −0.42]), when excluding those studies.

Three studies from China were included in the meta-analysis, and two of them were published in Chinese. Among them, one used an adequate dose (fluoxetine 20 mg) (39); one used an adequate but relative low dose (sertraline 50 mg) (53); and another one used a smaller dose (trazodone 50 mg) (50). Results indicated that when excluding those studies, antidepressants still were superior to placebo in terms of the rate of responder, reduction of target symptoms, and improvement of functional state, and inferior to placebo in terms of safety (withdraw from trails and the occurrence of patients with adverse events).

### Publication Bias

Results from Begg's funnel plot and the non-significant Egger's test (*p* = 0.30) showed no evidence for a potential publication bias for the rate of responder.

## Discussion

### Summary of Main Results

The meta-analysis included 31 RCTs totalling 2,340 participants with a mean treatment duration of 9.6 weeks across different subtypes of FGIDs. Although evidence has suggested that those syndromes overlap with one another, this is the first meta-analysis to break down the restrictions among different diagnostic labels of FGIDs. We found that antidepressants were superior to placebo in terms of efficacy and inferior with respect to tolerability. At post-treatment, the target gastrointestinal symptoms averagely had an improvement of 0.94 standardized units. Disability severity was significantly reduced and HRQoL was improved. 7.4 patients needed to be treated for one patient to respond or remiss, while about one in five treated with antidepressants experienced certain adverse effects. Common AEs experienced by patients taking antidepressants included nausea, sedation, dizziness, dry mouth, nervousness, diarrhea or constipation, headache, somnolence or insomnia, and abnormal ejaculation. Serious adverse events were only reported by IBS patients taking amineptine. Our findings were largely consistent with Jackson et al.'s study of the efficacy of antidepressants in treating FD, IBS or both, even though only data from 11 studies was pooled at that time (21). The efficacy was also comparable with antidepressants in treating acute major depression [NNT = 8.0 (95%CI 7.1 to 9.1)] (71), and with TCAs [NNT = 9 (range 7 to 16)] and SSRIs [NNT = 7 (range 7 to 8)] in treating depression in primary care settings (72). Unfortunately, the definition of NNH in treating depression varied, which made the comparison difficult.

### Subtypes of FGIDs

The efficacy of antidepressants did not differ significantly among different subtypes of FGIDs. Consistently, recent systematic reviews and meta-analysis indicated that antidepressants were effective for IBS (17, 18). Clinical reviews also supported the efficacy of antidepressants for patients with FD (10), functional esophageal disorders (19), and functional abdominal pain (20). Nevertheless, compared carefully, the studies included and the effect sizes in each review still differed. Take the recent meta-analyses of the effect of antidepressants in treating IBS for an example. This meta-analysis included 13 studies of patients with IBS, and 11 of them were shared with Kułak-Bejda et al.'s study (73). We additionally included two studies (56, 61), but did not include another seven studies due to following reasons: (1) two lacked the placebo as control group; (2) two included patients with general functional gastrointestinal disorder or functional bowel disorder, and were classified into the mixed subgroup in this meta-analysis (68, 69); (3) one treated patients with the combination of antidepressants and high-fiber diet, which was supposed to have possible therapeutic effect (62); (4) one with no available data before the washing period and the cross-over; (5) one with high risks of bias (35). Similar reasons also lead to the exclusion of certain studies that might be included in other systematic reviews (73–76). As a matter of fact, it should be noted that the study of Drossman et al. recruited patients “categorized as having IBS, painful functional constipation, chronic functional abdominal pain, and unspecified FBD” according to the Rome I diagnostic criteria (68). However, all other meta-analyses investigating IBS included this study without a special remark.

With different studies included and outcomes measured, different meta-analyses yielded varied effect sizes. According to our results, the NNT of antidepressants in treating FGIDs was 7.4 (95% CI 5.4 to 11.9), which was larger than the value of NNT in Jackson et al.'s study of FD, IBS or both [3.2 (95% CI 2.1 to 6.5)] (21), and in treating IBS (4 in Ford et al.'s study, and 4–5 in Ruepert et al.'s study) (17, 18). Besides the potential influence by including different studies, several other reasons might also account for the difference. For example, the definitions of responder/response rate varied across studies. As reported, this meta-analysis adopted relatively strict criteria, and the definitions of a responder in five studies included were similar with the definitions of a remission rate. Therefore, such criteria were likely to lead to a relatively small RR and large NNT. In addition, several studies included in this meta-analysis recruited only treatment refractory patients who have failed a regular therapy, like proton pump inhibitors (PPIs). As a result, the benefit of antidepressants has been very cautiously evaluated in this meta-analysis, if not underestimated.

Another challenge faced by meta-analyses in this topic was the changing definitions of FGIDs over time. For example, the newest Rome IV removed the term “discomfort” from IBS criteria and measured the existence of functional bowel disorders on a continuum as related to the degree of pain or the consistency of stool, as well as raised the threshold for symptomatic periods (77). Such changes are likely to lead to the migration of patients across categories over time. However, to which extent such changes would affect the efficacy of antidepressants in research remained unknown. Therefore, future studies investigating the efficacy of antidepressants for FGIDs based on the new Rome IV criteria are still needed. In addition, up to now, no RCT of antidepressants vs. placebo in treating patients with other common subtypes of FGIDs was available, such as nausea and vomiting disorders, and functional abdominal pain (defined as centrally mediated disorders of gastrointestinal pain in the Rome IV).

### Type of Antidepressants

Subgroup analyses confirmed the statistical significant benefit of TCAs (for the rate of responder), TeCAs (for both rate of responder and reduction of target symptoms), as well as SSRIs and SARI (for reduction of gastrointestinal symptoms). Evidence for the efficacy of SNRIs was inconclusive. According to a Cochrane review, SSRIs and TCAs were superior in improving global assessment, abdominal pain and symptom score in patients with IBS (14), but no other types of antidepressants was discussed. Among studies investigating antidepressants in treating major depressive disorder, a comparative meta-analysis showed that mirtazapine, escitalopram, venlafaxine, and sertraline were significantly more efficacious than other new-generation antidepressants, while reboxetine was less efficacious. Escitalopram and sertraline also demonstrated the best profile of acceptability (78). But another review found no major differences in both the rate of responder and the overall incidence of AEs across different types of second-generation antidepressants (79).

In the past, evidence for the superiority of any type of antidepressants was contradictory. Recommendations from the National Institute for Health and Care Excellence (NICE) on IBS in primary care recommended TCAs as second line treatment, and SSRIs only if TCAs are ineffective (80). Review for the treatment of FD recommended TCAs over SSRIs and SNRIs (10). Experts suggested selecting specific antidepressants according to the associated symptoms, such as a TCA or an SNRI for their advantages in reducing pain, an SSRI when there are dominant symptoms of anxiety/obsessive features/phobic behaviors, a TCA with diarrhea, an SSRI with constipation, and mirtazapine with nausea (81).

Actually, the rationale for antidepressants treatment in patients with FGIDs included the central effects of pain perception modulation and psychotropic action through increasing available neurotransmitters or regulating neuronal growth, as well as the peripheral influence on gastrointestinal motility and secretion and analgesic effects through the antagonistic effects on 5-HT1, 5HT2, 5-HT3, 5-HT4, 5-HT7, and H2 receptors (12, 81–83). Even though TCAs reduced pain sensitivity more effectively than SSRIs in chronic neuropathic animal models, and have gathered more clinical evidence from earlier time, there is no clear evidence for the overall advantages of TCAs over SSRIs in patients with FGIDs. The notable side effects of TCAs related to anticholinergic and antihistaminic actions and fatality after overdosed should also be taken into consideration. Furthermore, whether certain types of antidepressants are more effective for a specific symptom or syndrome requires further evidence.

### Dosage of Antidepressants

The NICE guidelines on IBS recommended to use TCAs at a low dose (80). Treatment of FD suggested beginning the antidepressants in modest dosages and increasing to an optimal level of benefit (81), and higher dosages may be needed if comorbid major depression is present. However, Jackson has criticized studies that used sub-therapeutic doses of antidepressants for somatic syndromes (84). According to our results, antidepressants at smaller dosages seemed to be less effective than those with adequate dosages, even though no significant difference was detected between them. Given consideration into the fact that only data from four studies of smaller dosages was available, and the psychological status of their participants was unclear, no definite conclusion could be drawn. Therefore, more evidence is needed to explore the efficacy and the mechanisms of low doses of antidepressants in treating FGIDs.

### Comorbid Depression

For long, the evidence for the benefit of antidepressant treatment in FGID patients with or without depression was limited and contradictory. For example, Ladabaum et al. reported that citalopram was not effective for non-depressed patients with IBS (59), but Drossman et al. found a more favorable response in IBS patients without depression (68). A review of functional chest pain suggested that the improvement in chest pain symptoms was independent of improvement in depression scores (85). However, most clinical guidelines have only recommended or justified the use of antidepressants in the presence of depression.

In our review, antidepressants were found to be effective in both the presence and absence of comorbid depression, even though the rate of responder for FGIDs patients without depression was inconclusive. However, the methodologies used in the original reviewed studies may have restricted our findings. For instance, approximately half of the studies in our meta-analysis included patients whether they were comorbid with depression or not, but they did not investigate whether the efficacy differed between both groups. The other half of studies used varied criteria, from standardized diagnostic tools to self-report questionnaires, to exclude depression. In summary, more studies using rigorous diagnostic tools to examine the extent to which the severity of depressive symptoms impacts the efficacy of antidepressants are needed to draw a conclusion.

### Treatment Duration

Generally, the acute period of antidepressants treatment of major depression disorder takes 8–12 weeks, while the full course therapy takes 6 to 12 months before decreasing the dose slowly. The NICE guideline for IBS also recommended continuing the treatment for 6 to 12 months or longer (70). However, only two RCTs included have followed up participants for more than 12 weeks. With limited evidence from studies with long-term follow-up, no significant difference was found regarding the efficacy of antidepressants at different endpoints in this meta-analysis. Future high-quality research is needed that follows participants over longer periods to elucidate how long would be enough for patients with FGIDs to recover, to remain stable, and to prevent relapse.

### Strengths and Limitations

Only double-blind randomized placebo-controlled trials with good study quality were included in this meta-analysis, representing a high level in evidence-based medicine. Research from China was systematically reviewed and added to the current evidence, as long as the defined quality standards were fulfilled; different subtypes of FGIDs were encompassed; and a couple of essential clinical queries were discussed through subgroup analyses.

Our review has several limitations. Firstly, only studies using antidepressants as treatment and placebo as comparison were included. The efficacy of antidepressants combined with antipsychotics or medications to ease pain, constipation, or dyspeptic symptoms has not been investigated. Trials that compared antidepressants with treatment as usual or other active medications were also excluded. Secondly, the safety data only included 11 studies that reported the number of patients with AEs. A large proportion of studies reported the frequency of AEs could not be synthesized. This could lead to biased estimation of the safety of antidepressants. Moreover, despite the efforts made as described in the methods section, it was difficult to find a way to synthesize data that was collected by different methods and measurements. For example, the definition of responders was actually close to remission rate in some original studies, so that the overall rate of responder could be estimated conservatively toward the benefit of antidepressants. Thirdly, the I^2^ statistic was high in most of our results, indicating that a large percentage of the heterogeneity between studies was not due to chance. However, our efforts to identify potential moderators failed. Fourthly, our findings about the efficacy of SNRIs, low doses of antidepressants, and antidepressants in intermediate to long term treatment should be interpreted with caution because of the scarcity of eligible evidence. In addition, the language was confined to English and Chinese. To which extent the results have been affected with the exclusion of possible studies published in German, French, Spanish or other language remained unknown. Lastly, the meta-analysis was not performed on the individual level, so that further exploration such as the difference between men and women was not possible. Even though the phenomenon that the majority of participants included were women corresponded to epidemiological data, to which extent the findings could be generalized to both men and women needs to be validated.

### Future Research Directions

Even though RCTs tended to define and restrict the diagnosis of participants as clear as possible, clinical studies showed that the overlap of different FGIDs was common. Therefore, our plea is that more studies should investigate the efficacy of antidepressants in FGIDs with a clear description of the comorbidity both within FGIDs and with a broader neurophysiologic pathology, such as fibromyalgia, chronic fatigue, chronic headache or general discomforts all over the body. In addition, RCTs of antidepressants vs. placebo in treating patients with functional bowel disorder based on the new Rome IV criteria are still needed, as well as patients with other common subtypes of FGIDs, such as nausea and vomiting disorders, and functional abdominal pain (defined as centrally mediated disorders of gastrointestinal pain in the Rome IV). Moreover, future high-quality research remains necessary to compare the efficacy of different types and dosages of antidepressants according to different clinical characteristics, to quantify the severity of gastrointestinal symptoms, to make clear diagnoses of the comorbidity of depression, and to follow participants over longer periods.

## Conclusion

Antidepressants were inferior to placebo in terms of tolerability (withdraw and the occurrence of adverse events) and superior regarding efficacy (reduction of target gastrointestinal symptoms, improving the functional status, and the overall rate of responder). The benefits of antidepressants must be balanced against the relatively high rates of adverse effects in the management of FGIDs.

## Data Availability Statement

The raw data supporting the conclusions of this manuscript will be made available by the authors, without undue reservation, to any qualified researcher.

## Author Contributions

All authors had full access to the data in this study and can take responsibility for the integrity of the data and the accuracy of the data analysis. NX and YD searched the database, retrieved the articles, rated the articles for quality, and abstracted the data independently. JW is the guarantor, who contributed to the conception of this study and was consulted if there were disagreements. RM contributed to the discussion of research topics and reviewed the manuscript. RL contributed to the study design, statistical analysis, data interpretation, as well as writing and revision of the manuscript.

### Conflict of Interest Statement

The authors declare that the research was conducted in the absence of any commercial or financial relationships that could be construed as a potential conflict of interest.
