# Supplementary material for: Antidepressants vs. Placebo for the Treatment of Functional Gastrointestinal Disorders in Adults: A Systematic Review and Meta-Analysis
Source: Front Psychiatry. 2018 Dec 4;9:659. doi: 10.3389/fpsyt.2018.00659 (PMC6288425; doi:10.3389/fpsyt.2018.00659)
Supplement: Supplementary file 1 [file Table_1.docx]

Search Strategies:

(functional gastrointestinal disorder* OR functional esophageal disorder* OR functional heartburn OR functional chest pain OR non-cardiac chest pain OR functional dysphagia OR globus OR functional gastroduodenal disorder OR functional dyspepsia OR non-ulcer dyspepsia OR belching disorder OR aerophagia OR nausea and vomiting disorder OR chronic nausea OR functional vomiting OR cyclic vomiting syndrome OR rumination syndrome OR functional bowel disorder OR irritable bowel syndrome OR functional bloating OR functional constipation OR functional diarrhea OR functional abdominal pain syndrome OR functional gallbladder and sphincter of Oddi disorder OR functional gallbladder disorder OR functional biliary sphincter of Oddi disorder OR functional pancreatic sphincter of Oddi disorder OR functional anorectal disorder OR functional fecal incontinence OR functional anorectal pain OR chronic proctalgia OR proctalgia fugax OR functional defecation disorder)

AND

(pharmacotherapy OR antidepressive agent OR tricyclic OR amitriptyline OR clomipramine OR dothiepin OR doxepin OR desipramine OR imipramine OR mianserin OR monoamine oxidase inhibitors OR moclobemide OR phenelzine OR serotonin uptake inhibitors OR citalopram OR fluoxetine OR paroxetine OR sertraline OR fluvoxamine OR serotonin and norepinephrine reuptake inhibitors OR duloxetine OR venlafaxine OR mirtazapine)

AND

(randomized controlled trail OR randomiz* OR randomiz* OR randomly NOT animals).

Supplementary Table 1. Overview of the randomized controlled trials in functional gastrointestinal disorders included within the meta-analysis (grouped by type of antidepressant)

| Antidepressant (maximum dose) | Syndrome | Depression excluded | N  (% female) | Age  (SD/ range) | Duration (weeks) | Drop-outs (%) | Country | Reference |
| --- | --- | --- | --- | --- | --- | --- | --- | --- |
| *Tricyclic antidepressants (TCA)* | |  |  |  |  |  |  |  |
| Amineptine (200 mg) | IBS | No | 40 (70.0) | 45.5 (8.4) | 6 | 10.0 | Greece | Alevizos 1989 |
| Amitriptyline (50 mg) | FD | Yes | 38 (60.5) | 40.0 (2.0) | 8 | 13.2 | Netherlands | Braak 2011 |
| Amitriptyline (75 mg) | IBS | No | 40 (50.0) | 35.0 (NS) | 12 | 45.0 | India | Rajagopalan 1998 |
| Amitriptyline (50 mg) | FD | Yes | 145 (74.5) | 44.0 (15.0) | 12 | 20.7 | USA | Talley 2015a |
| Amitriptyline (10mg) | IBS, diarrhoea | No | 54(38.9) | 36.0(12.0) | 8 | 14.8 | Iran | Vahedi 2008 |
| Desipramine (150 mg) | Functional bowel disorder | No | 216 (100) | 39.9 (12.1) | 12 | 23.6 | USA/ Canada | Drossman 2003 |
| Desipramine (150 mg) | Irritable colon syndrome | No | 44 (12.9) | 48.0 (18-76) | 8 | 29.5 | USA | Heefner 1978 |
| Doxepin (75 mg) | IBS | No | 50 (28.0) | 32.5 (15.5) | 6 | 12.0 | India | Vij 1991 |
| Imipramine (50 mg) | IBS | No | 107 (43.0) | 43.0 (13.1) | 12 | 47.6 | Lebanon | Abdul-Baki 2009 |
| Imipramine (25 mg) | Functional heartburn | No | 83 (80.7) | 49.0 (13.0) | 8 | 19.3 | Thailand | Limsrivilai 2016 |
| Imipramine (50 mg) | IBS | No | 26 (61.5) | NS | 12 | 25.0 | Australia | Talley 2008a |
| Mianserin (120 mg) | FGID | Yes | 49 (65.3) | 37.3 (12.5) | 7 | 4.1 | Norway | Tanum 1996 |
| Trimipramine (25mg) | IBS | No | 61(54.1) | 38.9 (13.2) | 4 | 0 | Norway | Myren 1982 |
| *Selective serotonin reuptake inhibitors (SSRI)* | |  |  |  |  |  |  |  |
| Citalopram (40 mg) | IBS | Yes | 54 (81.5) | 52.0 (14.0) | 8 | 16.7 | USA | Ladabaum 2010 |
| Citalopram (40 mg) | IBS | Yes | 23 (78.3) | 39.0 (3.0) | 6 | 8.7 | Belgium | Tack 2006 |
| Citalopram (40 mg) | IBS | No | 25 (60.0) | NS | 12 | 44.0 | Australia | Talley 2008b |
| Citalopram (20 mg) | Hypersensitive esophagus | No | 75 (NS) | NS | 24 | 0 | Greece | Viazis 2012 |
| Escitalopram (10 mg) | FD | Yes | 147 (75.5) | 44.0 (15.0) | 12 | 29.3 | USA | Talley 2015b |
| Fluoxetine (20 mg) | IBS | Yes | 40 (55.0) | 40.0 (2.0) | 6 | 15.0 | Netherlands | Kuiken 2003 |
| Fluoxetine (20mg) | Functional heartburn | No | 96(56.3) | 37.4(11.0) | 6 | 1.0 | Iran | Ostovaneh 2014 |
| Fluoxetine (20 mg) | IBS, constipation | No | 44 (61.4) | 34.9 (10.0) | 12 | 0 | Iran | Vahedi 2005 |
| Fluoxetine (20 mg) | NCCP | No | 81 (51.8) | 59.3 (10.8) | 4 | 1.2 | China | Zheng 2006 |
| Paroxetine (50 mg) | FCP | Yes | 50 (42.0) | 52.9 (10.6) | 8 | 14.0 | USA | Doraiswamy 2006 |
| Paroxetine (50 mg) | IBS | Yes | 72 (87.5) | 49.0 (18-75) | 12 | 19.4 | USA | Masand 2009 |
| Paroxetine (40 mg) | NCCP | No | 46 (47.8) | 57.4 (9.9) | 12 | 23.9 | Netherlands | Spinhoven 2010 |
| Sertraline (200 mg) | FCP | Yes | 30 (NS) | NS (18-85) | 8 | 16.7 | USA | Varia 2000 |
| Sertraline (50 mg) | FD | No | 193 (72.0) | 42.4 (NS) | 8 | 22.3 | China | Tan 2012 |
| Sertraline (200 mg) | NCCP | No | 58 (67.0) | 48.0 (12.0) | 34 | 15.5 | USA | Keefe 2011 |
| *Serotonin and norepinephrine reuptake inhibitors (SNRI)* | | |  |  |  |  |  |  |
| Venlafaxine (75 mg) | FCP | Yes | 50 (20.0) | 23.5 (1.9) | 4 | 2.0 | Korea | Lee 2010 |
| Venlafaxine (150 mg) | FD | No | 160 (60.0) | 52.0 (15.0) | 8 | 35.6 | Netherlands | Van Kerkhoven 2008 |
| *Noradrenergic and specific serotoninergic antidepressants (NaSSA)* | | | |  |  |  |  |  |
| Mirtazapine (15 mg) | FD | Yes | 34 (85.3) | 35.9 (2.3) | 8 | 11.8 | Belgium | Tack 2016 |
| *serotonin antagonist and reuptake inhibitor (SARI)* | | | |  |  |  |  |  |
| Trazodone (150 mg) | Esophageal symptoms | No | 29 (72.4) | 47.7 (3.7) | 6 | 20.7 | USA | Clouse 1987 |
| Trazodone (50mg) | FD | No | 80 (100.0) | 46.0 (10.5) | 6 | 0 | China | Liu 2007 |
| *Others* | | | |  |  |  |  |  |
| SJW (900 mg) | IBS | Yes | 70 (86.0) | 42.0 (NS) | 12 | 14.3 | USA | Saito 2009 |

Abbreviations: FCP = functional chest pain; FD = functional dyspepsia; IBS = irritable bowel syndrome; NCCP = non-cardiac chest pain; NS = not stated.
